# First Report on *Choanephora cucurbitarum* Causing Choanephora Rot in *Chenopodium* Plants and Its Sensitivity to Fungicide

**DOI:** 10.3390/jof9090881

**Published:** 2023-08-28

**Authors:** Hui Yin, Miao Tian, Yufei Peng, Nan Qin, Hong Lü, Lu Ren, Xiaojun Zhao

**Affiliations:** College of Plant Protection, Shanxi Agricultural University, Taiyuan 030031, China

**Keywords:** djulis, fungal, quinoa, sporangiola, sporangiospore, soft rot

## Abstract

Choanephora rot of *Chenopodium* plants (CRC) was observed at the flowering stages in seven plantations of Shanxi Province, China. CRC had caused leaf, stem, and panicle neck rot of *C. quinoa*, panicle neck and stem rot of *C. formosanum*, and stem rot of *C. album*. Typical symptoms included water-soaked, rapid soft rotting, and abundant sporulation on the whole panicle necks, stems, and leaves. Based on morphological characteristics, phylogenetic analyses, and pathogenicity tests, the pathogens were identified as *Choanephoraceae cucurbitarum*. Sporangiola and sporangiospore of *C. cucurbitarum* germinated at 30 °C and were able to germinate by two h post-inoculation (hpi). The germination rates of sporangiola and sporangiospore significantly increased at 3 to 4 hpi, and the germination rates ranged from 91.53 to 97.67%. The temperature had a significant effect on the pathogenicity of *C. cucurbitarum* the optimum pathogenic temperatures for stems of *C. quinoa*, *C. formosanum* and *C. album* were 30 °C after one day post-inoculation. *Choanephoraceae cucurbitarum* could infect white and red quinoa panicle necks between 20 and 30 °C, and the average lesion lengths were 0.21 to 3.62 cm. Among the five tested fungicides (boscalid, dimethomorph, isopyrazam, propiconazole, and tebuconazole), isopyrazam showed higher sensitivity to sporangiola germination of *C. cucurbitarum*, with an EC_50_ value of 0.6550 μg/mL. Isopyrazam and tebuconazole strongly inhibited the sporangiospore germination of *C. cucurbitarum*, which showed EC_50_ values of 0.4406 and 0.3857 μg/mL. To our knowledge, the present study found for the first time that *C. cucurbitarum* is a pathogen causing panicle neck of *C. formosanum* and stem rot of *C. formosanum* and *C. album*, while CRC first appeared in the quinoa panicle necks, and gradually expanded to stems and leaves.

## 1. Introduction

The genus *Chenopodium* which includes more than 170 species, has been gathered as grains, potherbs, and weeds at various times and places throughout human history [1,2,3,4,5,6]. Nowadays, *Chenopodium* plants such as *C. quinoa*, *C. formosanum*, and *C. album* are recognized as excellent sources of nutrients, amino acids, and vitamins from their grains and leaves [6,7,8,9]. With the increasing popularity of *C. quinoa* and *C. formosanum*, China has invested in the large-scale production of this crop [3,9,10,11].

As with any crop, yield and quality may be impacted by pathogenic organisms. Among the *Chenopodium* plants, quinoa disease is probably the most notable. The most severe fungal diseases of quinoa included panicle rot caused by *Alternaria alternata*, *Fusarium citri*, and *Trichothecium roseum* [12]; gray mold caused by *Botrytis cinerea* [13]; stem rot and black stem caused by *Choanephora cucurbitarum* and *Ascochyta caulina* [14,15]; and leaf spot caused by *Cercospora* cf. *chenopodii* [16] and *Heterosporicola beijingense* [17]. Comparatively, the diseases of *C. formosanum* and *C. album* have received less attention. There is little known about the disease of *C. formosanum*. The diseases of *C. album* were mainly leaf spot caused by *A. alternata* [18], *C.* cf. *chenopodii* [16,19], *F. equiseti* [20], and *Nigrospora pyriformis* [21].

*Choanephora* is classified in *Choanephoraceae* (*Mucorales*, *Mucoromycota*). Currently, the genus *Choanephora* includes only two accepted species (*C. cucurbitarum* and *C. infundibulifera*), which are recognized in MycoBank (http://www.mycobank.org, 2023). *Choanephora* species are destructive pathogens and mainly reported as the causal agents of seedling rot of castor [22], shoots tips of green bean and pepper [23], flower blight [24,25,26,27,28,29,30], leaf and stem rot [30,31,32,33,34], and fruit rot [23,24,35,36]. Previous studies have shown that *C. cucurbitarum* has a wide host range with reports on 25 host species (Table 1). However, little is known about the damage of *C. cucurbitarum* to *Chenopodium* plants worldwide. In 2018, Sun et al. reported that *C. cucurbitarum* could infect quinoa stems (Table 1). There was no systematic report that *C. cucurbitarum* caused the rot of the panicle neck and leaf of *Chenopodium* plants.

When CRC incidences are severe, chemical control is one of the important measures. However, little research has been performed on the sensitivity of fungicides to pathogens of CRC. The toxicological effects of different types of fungicides are different, resulting in different control effects [47]. Therefore, measuring the sensitivity of pathogens to fungicides will help to control the CRC. The present study aimed to identify the species causing CRC based on morphology traits, molecular phylogenetic analysis, and pathogenicity. Our results would provide a comprehensive understanding of CRC to improve the recognition and prevention of the disease.

## 2. Materials and Methods

### 2.1. Sampling and Pathogen Isolation

Between July and August 2022, CRC were observed on many plantations in five regions of Shanxi Province, namely Jingle (Latitude: 38.2498 N; Longitude: 111.8926 E), Taigu (Latitude: 37.4316 N; Longitude: 112.5847 E), Wutai (Latitude: 38.8901 N; Longitude: 113.5118 E), Xinzhou (Latitude: 38.4669 N; Longitude: 112.7251 E), and Yuanping (Latitude: 38.7775 N; Longitude: 112.7345 E). We collected *Chenopodium* plants with typical symptoms, having water-soaked and soft rot on the panicle necks, stems, and leaves. Fresh samples were the basic biological material for study. Therefore, samples were brought back to the laboratory and stored at 4 °C for further examination. Samples were randomly collected from these five counties and about 23 samples were collected. In addition, we investigated the incidence and yield loss of CRC in the field. Incidence was determined as a percentage of visual CRC symptoms on quinoas of total number of quinoas. At harvest ripeness, yield loss was estimated based on yield of diseased field and no yield loss field. 

Samples with monosporous sporangiola were selected and were cut into small pieces (1 × 1 cm). To obtain the pathogens, monosporous sporangiola were directly picked from the small pieces showing typical symptoms using a stereomicroscope and cultured on potato dextrose agar (PDA) (Solarbio, Beijing, China) in a climate chamber (fluorescent cycle of 12 h light/12 h dark) at 25 °C for 1 day [27,48]. Then, pure isolates were obtained using the single-mycelium tipping method on PDA and stored at 4 °C [27,49]. Morphological characteristics were used to select the representative isolates at random from all isolates for continued assessment. A total of 15 pure isolates with identical morphological characteristics were obtained, and five were randomly selected for morphology, molecular identification, and pathogenicity test. 

### 2.2. Morphological Analysis

The representative isolates were cultured on PDA in a climate chamber (fluorescent cycle of 12 h light/12 h dark) at 25 °C for 1–3 days. The colony diameters were measured using the cross intersection method after 1 day [50]. Cultural features, including colony morphology and color, were also observed at 2 days.

The representative isolates were cultured on PDA at 25 °C for 2 days until sporangiola formed. The microscopic features of sporangiola were directly observed on PDA using an SMZ18 stereomicroscope (Nikon, Tokyo, Janpan). Microscopic structures of sporangiola were examined using a BX53 microscope (Olympus, Tokyo, Janpan) [27]. In order to view the detailed structures of sporangia and sporangiospore, the representative isolates (LMJM-2, LMJM-3, LMJM-5, LMJM-7, and LMJM-9) were, respectively, cultured on oatmeal agar (OA) (Maokang, Shanghai, China) and incubated at 25 °C for 7 days. The detailed structures of sporangia and sporangiospore were observed and measured using an SMZ18 stereomicroscope and BX53 microscope, respectively. For each representative isolate, the sizes of 50 sporangiola, sporangiophores, sporangia, and sporangiospores were randomly measured and recorded. 

### 2.3. Molecular Identification

For DNA extraction, the representative isolates were cultured on PDA and incubated at 25 °C for 3 days. Mycelia were scraped from PDA, and then ground in liquid nitrogen. Genomic DNA was extracted using an Ezup column fungi genomic DNA purification kit (Sangon Biotech, Shanghai, China) following the manufacturer’s protocol. The large subunit region (LSU) and internal transcribed spacer region (ITS) were amplified using the primer pairs LROR/LR7 and ITS1/ITS4 [51]. The PCR amplification procedures for LSU and ITS were as follows: initial denaturation at 95 °C for 5 min, followed by 35 cycles of denaturation at 95 °C for 90 s, annealing at 55 °C for 90 s, extension at 72 °C for 1 min, and a final extension at 72 °C for 10 min. The PCR amplification products were separated using 1% agarose gel electrophoresis, and the products were purified using a QIAquick Gel Extraction kit (Qiagen, Inc., Valencia, CA, USA). The PCR products were sent to Sangon Biotech (Shanghai, China) Co., Ltd. for sequencing to obtain the sequences, and uploaded to GenBank. *Blakeslea trispora* (CBS 564.91) was used as the outgroup for the phylogenetic tree. The maximum likelihood (ML) method was performed using PAUP (v. 4.0b10) with 1000 bootstrap replicates based on the LSU and ITS gene sequences [52]. Details of the sequences used for phylogenetic analysis are provided in Table 2.

### 2.4. Sporangiola and Sporangiospore Germination

Sporangiola and sporangiospore of the representative isolates were, respectively, collected from PDA and OA. Then, spore suspensions of sporangiola and sporangiospore were, respectively, prepared at a concentration (1 × 10^5^ cfu/mL) with sterile distilled water. The PDA temperature was at ~50 °C, and 200 μL of the PDA was applied to the sterile microscope slides (26 × 76 mm) [15]. After PDA solidification, the suspension (20 μL) was inoculated on the ready-prepared microscope slide and incubated in a desiccator with a relative humidity (RH) of 75% (saturated NaCl saline solution) at 30 °C. After 1, 2, 3, and 4 h, the morphologies of sporangiola and sporangiospore germination were observed using a BX53 microscope, and counted to determine the germination rate from 200 spores in each of the three replicates.

### 2.5. Pathogenicity Tests

The pathogenicity of all representative isolates (LMJM-2, LMJM-3, LMJM-5, LMJM-7, and LMJM-9) was assessed on healthy plants of *Chenopodium quinoa*, *C. formosanum*, and *C. album*. *Chenopodium quinoa* (white quinoa: Jingli No. 1, red quinoa: Jingli No. 3), *C. formosanum* (Xinli No. 1), and *C. album* were cultivated in the greenhouse from seeds until the flowering stage (fluorescent cycle of 12 h light/12 h dark). To determine the pathogenicity of sporangiola on the panicle necks of *C. quinoa* and *C. formosanum*, the panicle necks were rinsed with sterile distilled water several times and then air-dried. Then, the sterile cotton wools were immersed in the prepared sporangiola suspension (~200 μL, 1 × 10^5^ cfu/mL) and inoculated on panicle necks [27]. The representative isolates were inoculated on 5 plants (one plant per pot). The control plants were treated in the same way with sterile distilled water. Each treatment was administered 3 times and conducted twice. Before inoculation stems of *C. quinoa*, *C. formosanum*, and *C. album*, the stems were rinsed with sterile distilled water several times and then air-dried. Sporangiola suspension (1 × 10^5^ cfu/mL) was inoculated on stems, as previously described. The representative isolates were inoculated on 5 plants. The control plants were inoculated in the same way with sterile distilled water. Each treatment consisted of three replicates and the experiment was conducted twice. In addition, the quinoa leaves were surface sterilized, and 0.5 mL of the sporangiola suspension (1 × 10^5^ cfu/mL) was inoculated on the leaf surface using a sterile handheld sprayer. Control leaves were inoculated in parallel using sterile distilled water. Each treatment was applied to three leaves and repeated five times. After inoculation, all inoculated and control plants described above were incubated in a climate chamber at 30 °C and RH = 75 ± 2%, with a 12 h photoperiod. The symptoms were monitored and recorded over 1–3 days, until the experiments were completed. 

To measure the effect of temperature on infection, we used a sporangiola suspension (1 × 10^5^ cfu/mL) inoculated on the stems of *C. quinoa*, *C. formosanum*, and *C. album* (10 plants per replicate) and panicle necks of white and red quinoa (10 plants per replicate). The control group was inoculated similarly with sterile distilled water. The experiment was conducted twice, and each treatment consisted of three replicates. After inoculation, all inoculated and control plants were placed in a climate chamber (RH = 75 ± 2%, 12 h photoperiod) with a temperature gradient of 10, 15, 20, 25, and 30 °C. The lesion lengths on the stem and panicle neck were measured after 3 days post-inoculation (dpi). To confirm Koch’s postulates, pathogens were reisolated and reidentified from symptomatic panicle necks, stems, and leaves of all inoculated plants.

### 2.6. Sensitivity of Sporangiola and Sporangiospore Germination to Five Fungicides

In order to identify the inhibition activity of fungicides on the germination of spores (sporangiola and sporangiospores) of *C. cucurbitarum*, we screened 5 fungicides. Boscalid (97.0%), dimethomorph (98.5%), isopyrazam (95.0%), propiconazole (95.4%), and tebuconazole (97.3%) were, respectively, dissolved in acetone to prepare 1 × 10^4^ μg/mL stock solutions [47,53]. The stock solutions of five fungicides were diluted into serial dilutions using sterile distilled water and added to PDA at ~50 °C to prepare the fungicide-containing PDAs [15,53] (Table 3). Preliminary testing showed that acetone was less than 0.25%; this did not affect the sporangiola and sporangiospore germination. Therefore, the same volume of acetone was added to PDA as a blank control. 

Different serial dilutions of the fungicide-containing PDAs were prepared (Table 3); 200 μL of each of the fungicide-containing PDAs was applied to the sterile microscope slides (26 × 76 mm) [15]. Spore suspension (20 μL, 1 × 10^5^ cfu/mL) was inoculated onto the ready-prepared microscope slide after agar solidification and incubated in a desiccator at 25 °C and RH = 75%. Spore germination was, respectively, counted to determine the germination inhibition rates after 4 h [15,53]. The experiment was performed twice, and each fungicide treatment and control contained three replicates.

The log transformation of the each treatment fungicide concentration represented the independent variable (X) and the probability of the corresponding germination inhibition rate represented the dependent variable (Y). With the regression equation, the EC_50_ value with a 95% confidence level to each treatment fungicide was determined [47,54].

### 2.7. Data Statistics and Analysis

Data were analyzed with SPSS statistics 19.0 by one-way ANOVA, and means were compared using Tukey’s test at a significance level of *p* = 0.05. Letters indicate significant differences (*p* = 0.05). 

## 3. Results

### 3.1. Field Symptoms

**Figure 1:**
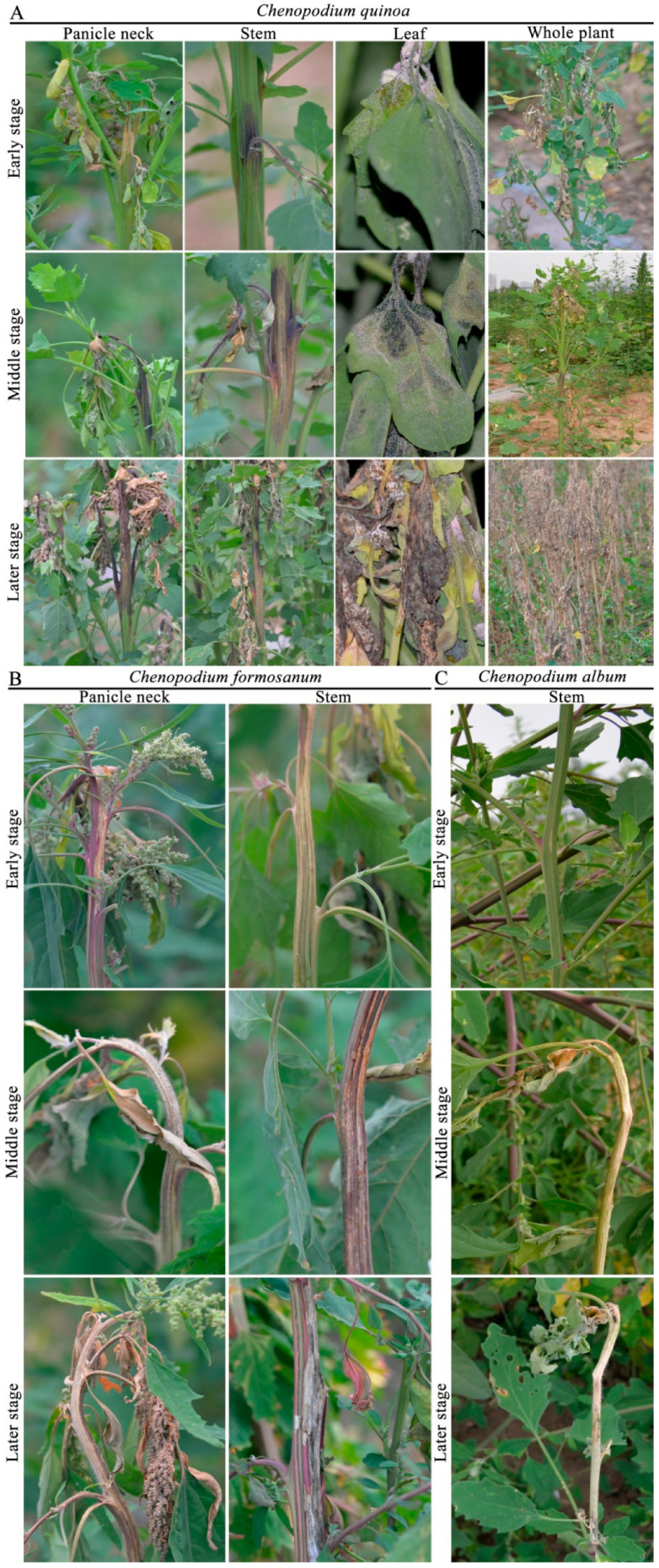
Symptoms of Choanephora rot on panicle necks, stems, and leaves of *Chenopodium quinoa* (**A**) panicle necks and stems of *C. formosanum* (**B**) and stems of *C. album* (**C**).

Choanephora rot of *Chenopodium* plants (CRC) primarily infected panicle necks, stems, and leaves. The incidence of CRC was approximately 65%, and the yield of *C. quinoa* and *C. formosanum* might decrease by over 80% in the fields where diseases were the most severe in Jingle, Taigu, and Xinzhou of Shanxi Province. These diseased panicle necks, stems, and leaves were usually discoloured, water-soaked, and soft rotted (Figure 1). Interestingly, CRC first infected the quinoa panicle necks, and then gradually spread towards to the stems and leaves (Figure 1A). The initial symptoms began as pale to tan lesions, and the margins between the lesions and healthy tissues were clear (Figure 1A). Subsequently, the color of the lesions on quinoa turned brown to black and water-soaked, resulting in rapid soft rotting of the whole panicle necks (Figure 1A). In the later stages, abundant sporulation occurred along the panicle necks, and then encompassed the entire panicles, resulting in quinoa panicles being blighted (Figure 1A). When CRC infected the quinoa stems, it primarily appeared in the middle and lower branches of the main stems (Figure 1A). Symptoms on the quinoa stems consisted of brown to black coloring and water-soaking, and followed by rapid soft rot (Figure 1A). Symptoms on quinoa leaves first developed on petioles resulting in wilting and rotting, and then expanded to leaves. Initial symptoms on the base of leaves appeared as water-soaked and darkgreen. A soft rot developed together with abundant sporulation and led to quinoa leaves’ blight (Figure 1A).

Additionally, CRC primarily infected the panicle necks and stems of *C. formosanum*, and did not usually infect the leaves (Figure 1B). Stems symptoms on *C. formosanum* appeared pale to grayish, with necrotic lesions, and they were covered with masses of sporangiola (Figure 1B). In contrast, stem symptoms on *C. album* initially consisted of pale to tan necrotic lesions, resulting in the infected stems breaking off the rest of the plant (Figure 1C).

### 3.2. Morphological Characteristiscs of the Choanephora cucurbitarum

**Figure 2:**
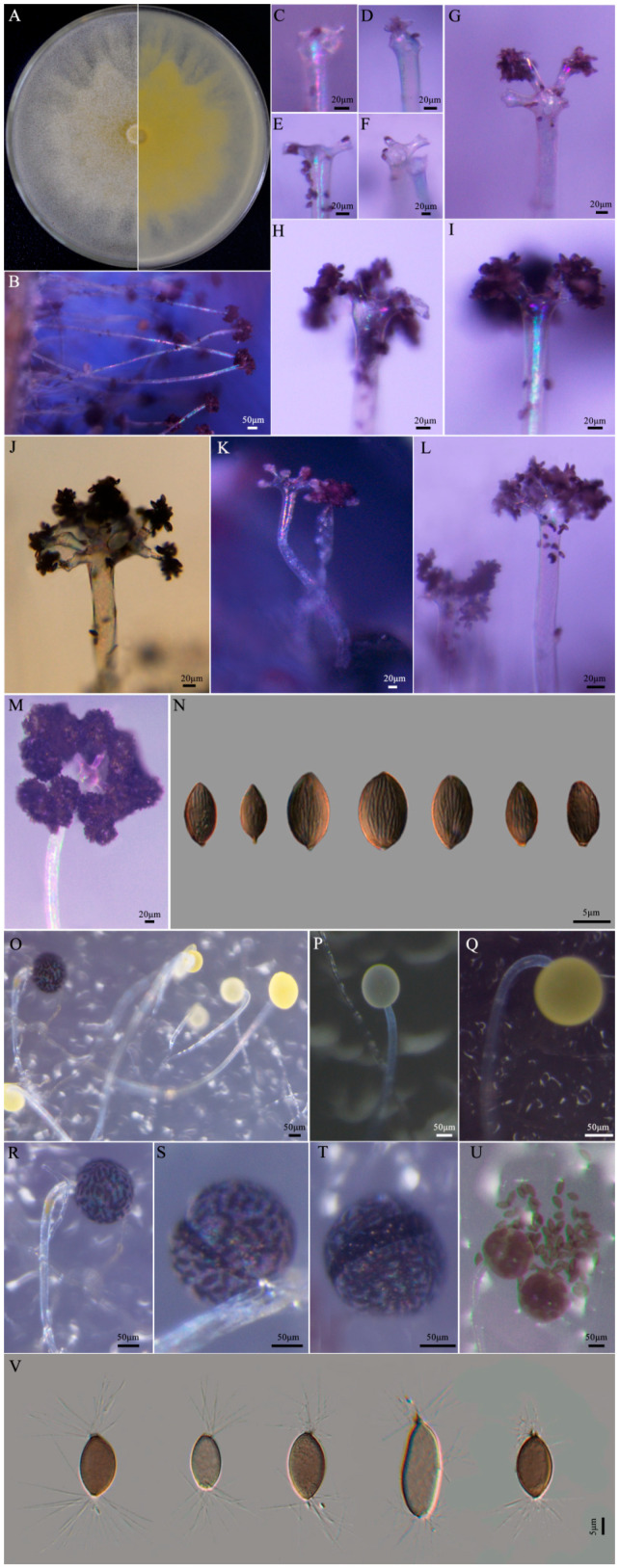
Morphological characteristics of *Choanephora cucurbitarum* from *Chenopodium* plants. (**A**) Colony on PDA for 2 days, (**B**) sporangiophores bearing sporangiola, (**C**–**G**) sporangiophores with apically dilated and bearing secondary vesicles, (**H**,**I**) sporangiola readily detached at maturity, (**J**–**L**) secondary vesicles with a head of sporangiola, (**M**) mature sporangiola with mulberry-like head, (**N**) sporangiola with longitudinal striation, (**O**) sporangiophores bearing sporangia, (**P**,**Q**) pale yellow to yellow sporangium, (**R**) sporangiophore bearing mature sporangium, (**S**,**T**) dehiscent sporangium, (**U**) sporangiospores from sporangia, and (**V**) sporangiospores with appendages.

The colonies grew rapidly on PDA, reaching 74–76 mm diameters in one day. After two days, colonies were white and cottony, with scattered monosporous sporangiola, and appearing pale yellow from below (Figure 2A).

Abundant sporulation of *C. cucurbitarum* could be observed in the infected stems (Figure 2B). Sporangiophores bearing sporangiola were hyaline, aseptate, slightly curved, and 362.4–2138.1 × 8.4–31.7 μm in size (mean = 1384.3 × 20.4 μm) (Figure 2B). Sporangiophores apically dilated to form a primary vesicle, from which secondary vesicles were produced (Figure 2C–G). The secondary vesicles bore sporangiola and readily detached at maturity, leaving a clathrate structure (Figure 2H–J). The primary vesicles of sporangiophores had risen to stalks terminating into secondary vesicles, each stalk bearing a head of mature sporangiola (Figure 2K,L). Sporangiophores bearing mature sporangiola had mulberry-like heads (Figure 2M). Monosporous sporangiola were brown to dark brown, ellipsoid to broadly ellipsoid, subtended by a short cylindrical pedicel, distinctly longitudinally coarsely striate, and 12.2–19.4 × 7.5–12.2 μm in size (mean = 15.0 × 9.7 μm) (Figure 2N).

Sporangia could be observed on OA (Figure 2O). Sporangiophores were aseptate, hyaline, nonbranching, bearing sporangium in a nodding fashion, and 68.8–828.8 × 7.3–28.4 μm in size (mean = 351.1 × 14.8 μm) (Figure 2O). Sporangia were often pale yellow to yellow initially but brown to intense black at maturity, and globose to subglobose (Figure 2P–R). Mature sporangia were tuberculate, 41.8–167.4 μm in diameter (mean = 98.6 μm), and dehiscent, which allowed the release of sporangiospores (Figure 2S–U). Sporangiospores from sporangia were brown, fusiform to elliptical at each pole with >10 hyaline appendages, and 13.2–23.9 × 6.7–12.8 μm in size (mean = 19.2 × 9.5 μm) (Figure 2V).

### 3.3. Phylogenetic Analysis of the Choanephora cucurbitarum

The sequence lengths of LSU and ITS from the representative isolates were 667 and 534 bp, respectively. All sequences of the representative isolates (LMJM-2, LMJM-3, LMJM-5, LMJM-7, and LMJM-9) were submitted to GenBank (Table 2). 

**Figure 3:**
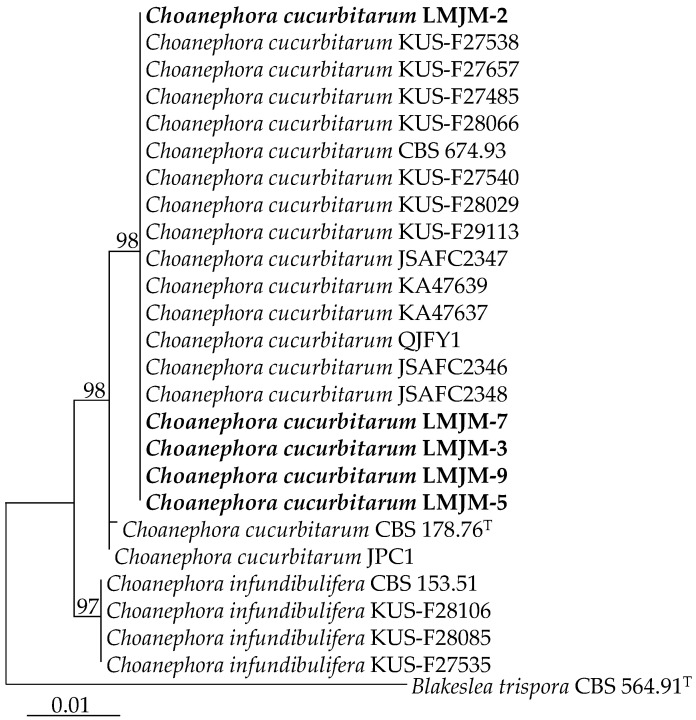
Phylogenetic tree of *Choanephora cucurbitarum* isolates (LMJM-2, LMJM-3, LMJM-5, LMJM-7, and LMJM-9) from *Chenopodium* plants and their related isolates based on LSU and ITS sequences using the maximum likelihood (ML) method. Ex-type strains were indicated with (T) in the end of the taxa labels, and our strains are in bold.

A phylogenetic tree was constructed using *Blakeslea trispora* (CBS 564.91^T^) as the outgroup. The results showed that the representative isolates (LMJM-2, LMJM-3, LMJM-5, LMJM-7, and LMJM-9) clustered in the same branch as sixteen isolates of *C. cucurbitarum* (KUS-F27538, KUS-F27657, KUS-F27485, KUS-F28066, CBS 674.93, KUS-F27540, KUS-F28029, KUS-F29113, JSAFC2347, KA47639, KA47637, QJFY1, JSAFC2346, JSAFC2348, CBS 178.76^T^, and JPC1) with a 98% bootstrap support rate, indicating that the representative isolates were the closest relationship with *C. cucurbitarum* (Figure 3). 

### 3.4. Sporangiola and Sporangiospore Germination of Choanephora cucurbitarum

**Figure 4:**
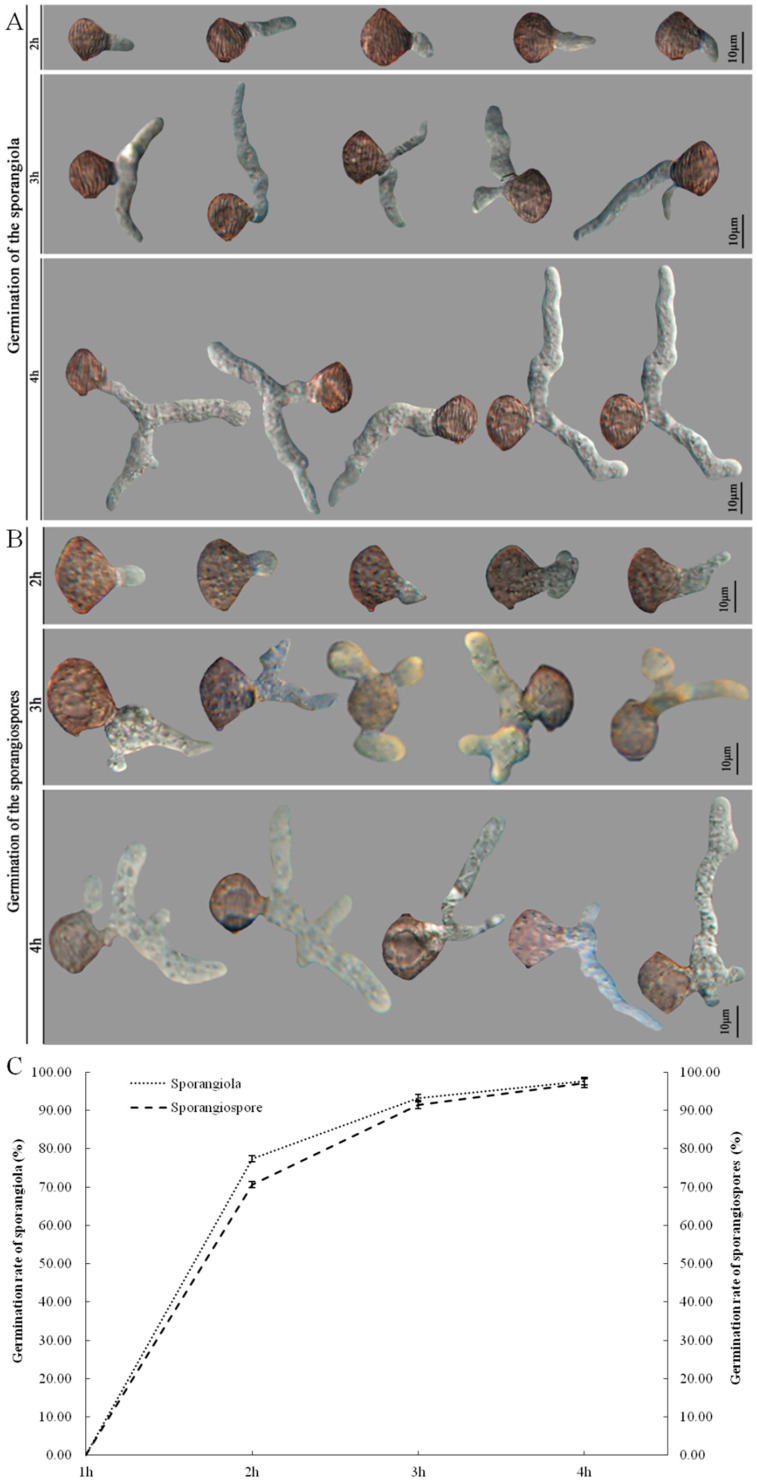
Germination of the sporangiola (**A**) and sporangiospores (**B**) of *Choanephora cucurbitarum* at 2, 3, and 4 h (**C**).

Sporangiola and sporangiospores of *C. cucurbitarum* were germinated at 30 °C, and the morphology of sporangiola and sporangiospore germination were separated at two representative stages of germ tubes formation and germ tubes elongation. At the stage of germ tubes formation, the germ tubes were able to germinate from the central part of the sporangiola and sporangiospore by 2 h post-inoculation (hpi) (Figure 4A,B). The mean germ tube lengths of sporangiola and sporangiospores were 11.77 and 8.95 μm. At the stage of germ tubes elongation, the branches of germ tubes appeared and the mean germ tube lengths of sporangiola and sporangiospores were 23.90–39.26 μm and 25.62–54.13 μm by 3–4 hpi. Germination rates of sporangiola and sporangiospores were 77.43% and 70.67% at 2 hpi. The germination rates of sporangiola and sporangiospores significantly increased at 3–4 hpi, and the germination rates ranged from 91.53 to 97.67%, and the differences were not significant (Figure 4C).

### 3.5. Pathogenicity Analysis of Isolates LMJM-2, LMJM-3, LMJM-5, LMJM-7, and LMJM-9

**Figure 5:**
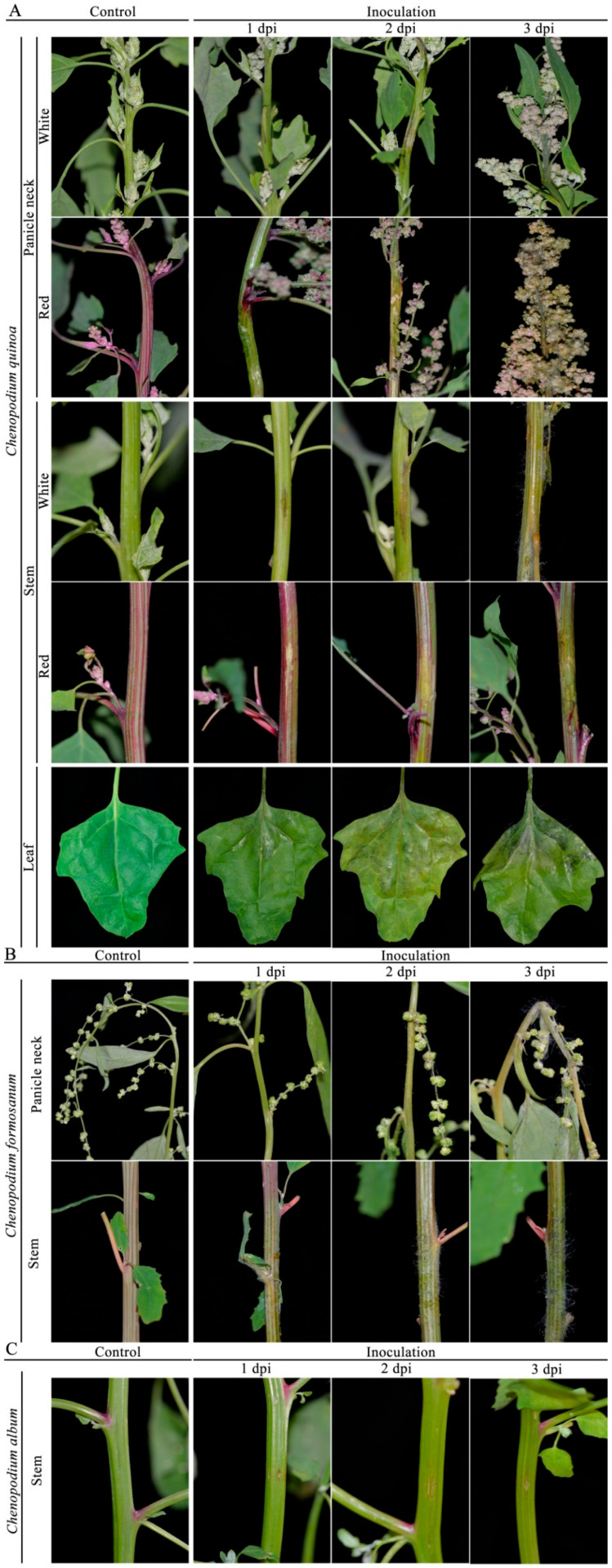
Symptoms on *Chenopodium quinoa* (**A**), *C. formosanum* (**B**), and *C. album* (**C**) induced by inoculation of representative isolates (LMJM-2, LMJM-3, LMJM-5, LMJM-7, and LMJM-9) of *Choanephora cucurbitarum*.

Pathogenicity tests showed that *C. cucurbitarum* could infect quinoa panicle necks, stems, and leaves. No symptoms were observed in the control groups (Figure 5A). One day after inoculation, pale brown necrosis lesions were found at the inoculation sites of white quinoa panicle necks, and the margins between the lesions and healthy tissues were obvious. By comparison, the inoculation red quinoa panicle necks were greyish-white, water-soaked, and soft rotted. At 2 dpi, the lesions further enlarged, and the lesions’ lengths ranged from 5.71 to 6.29 cm. Noticeably, the lesions on white quinoa were brown to black, water-soaked, and soft rotted. At 3 dpi, obvious CRC symptoms that were identical to the naturally infected panicle necks were observed (Figure 5A). The color of the lesions on white and red quinoa stems induced by *C. cucurbitarum* were different. At 1 dpi, obvious black and water-soaked lesions were found on the white quinoa stems; however, pale brown necrosis lesions were found on the red quinoa stems. With the development of disease, the typical symptoms developed on inoculated stems and were covered with masses of sporangiola at 3 dpi (Figure 5A). In addition, pathogenicity tests of *C. cucurbitarum* were performed on the quinoa leaves. At 1 dpi, obvious black and water-soaked lesions were found on the quinoa petioles and leaves. The color of the diseased leaves gradually became dark green, with a film of mold, resulting in rapid soft rotting of the whole (Figure 5A). There were no symptoms in the control (Figure 5A).

The pathogenicity tests of *C. cucurbitarum* were further inoculated on the panicle necks and stems of *C. formosanum* and stems of *C. album*. Three days after inoculation, obvious symptoms appeared on the panicle necks and stems of *C. formosanum* and stems of *C. album*, the inoculated panicle necks and stems were covered with masses of sporangiola. The control plants of *C. formosanum* and *C. album* remained healthy (Figure 5B,C). *C. cucurbitarum* was reisolated from the panicle necks, stems, and leaves that showed symptoms, and their reidentification was confirmed by morphology and molecular characterizations, as described above. Collectively, the morphology, molecular characterization, and pathogenicity confirmed that *C. cucurbitarum* was the causal agent of CRC.

### 3.6. Effect of Temperature on the Pathogenicity of Choanephora cucurbitarum

**Figure 6:**
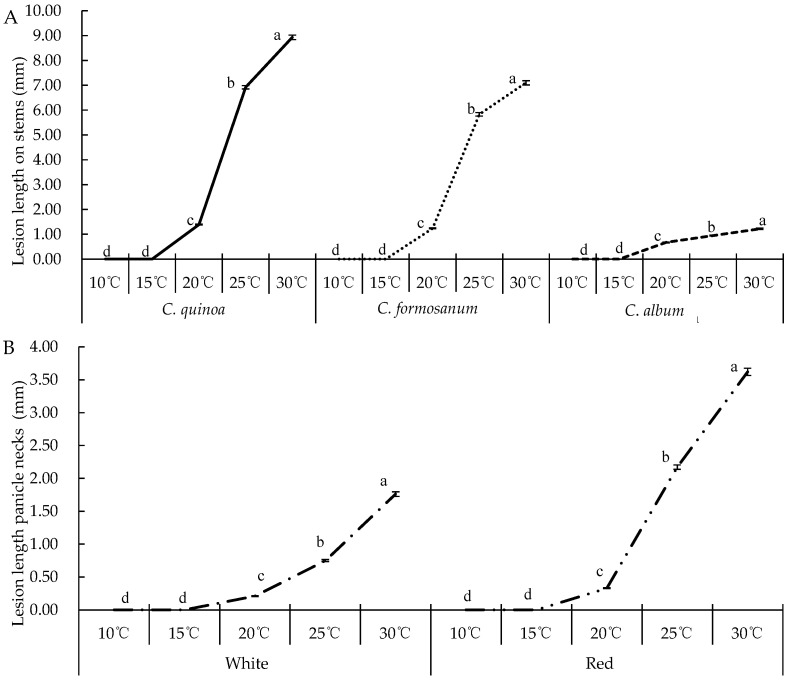
Effect of temperature on pathogenicity of the representative isolates of *Choanephora cucurbitarum*. (**A**) Pathogenicity on stems of *C. quinoa*, *C. formosanum*, and *C. album* at different temperatures, (**B**) lesions lengths on panicle necks of white and red quinoa that were inoculated with *Choanephora cucurbitarum* and incubated at different temperatures. Data were analyzed with SPSS statistics 19.0 by one-way ANOVA, and means were compared using Tukey’s test at a significance level of *p* = 0.05. Different letters indicate significant differences (*p* = 0.05).

Temperature had a significant effect on the pathogenicity of *C. cucurbitarum* (Figure 6). *Choanephora cucurbitarum* could infect the stems of *C. quinoa*, *C. formosanum*, and *C. album* between 20 and 30 °C. The optimum pathogenic temperature for stems of *C. quinoa*, *C. formosanum*, and *C. album* was 30 °C, and the lesions lengths were 8.93, 7.10, and 1.22 cm, respectively. When the temperatures were below 15 °C, there were no lesions in all stems (Figure 6A).

*Choanephora cucurbitarum* could infect white and red quinoa panicle necks between 20 and 30 °C, and the average lesions lengths were 0.21–3.62 cm. The optimal pathogenic temperature of *C. cucurbitarum* was 30 °C, and the lesions lengths were 1.76 cm and 3.62 cm, which were significantly higher than other treatments. When the temperature was at 20 °C, the lesions lengths were significantly reduced to 0.21 cm on white quinoa and 0.33 cm on red quinoa. At 10 and 15 °C, the lesions lengths were 0 cm (Figure 6B).

### 3.7. Effect of Five Fungicides on Spore Germination of Isolate LMJM-2

**Figure 7:**
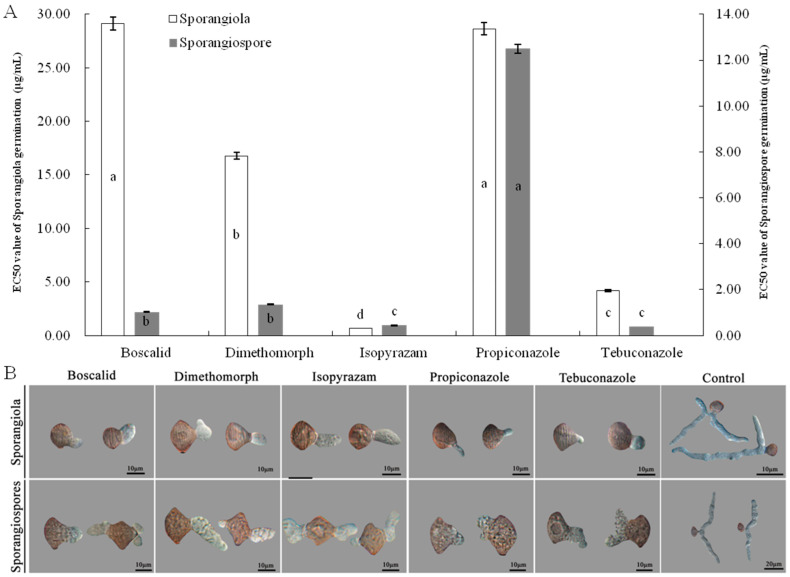
Effects of five fungicides on germination of sporangiola and sporangiospores of isolate LMJM-2. (**A**) Inhibition activities of five fungicides on germination of sporangiola and sporangiospores, (**B**) morphology of germ tubes of sporangiola and sporangiospores treated with five fungicides at EC_50_. Different letters indicate significant differences (*p* = 0.05).

The spores (sporangiola and sporangiospore) of *C. cucurbitarum* showed different sensitivity to five fungicides. Isopyrazam was found to be the most effective fungicide against sporangiola germination of *C. cucurbitarum*, with an EC_50_ value of 0.6550 μg/mL, and the differences compared with the other four fungicides were significant. Furthermore, the EC_50_ values of boscalid, dimethomorph, propiconazole, and tebuconazole were 29.1273, 16.7763, 28.6449, and 4.1957 μg/mL, respectively (Figure 7A). Among the five fungicides, those that most strongly inhibited the sporangiospore germination of *C. cucurbitarum* were isopyrazam and tebuconazole, which showed EC_50_ values of 0.4406 and 0.3857 μg/mL. The differences between isopyrazam and tebuconazole were not significant, but the differences compared with the other three fungicides were significant. Moderate inhibitory effects on the sporangiospore germination were boscalid and dimethomorph, which showed EC_50_ values of 1.0250 and 1.3493 μg/mL. In contrast, propiconazole showed a low inhibitory effect against the sporangiospore of *C. cucurbitarum*, with an EC_50_ value of 12.4997 μg/mL (Figure 7A). 

As in the control treatment, when cultured on PDA-containing fungicide, the sporangiola and sporangiospore germinations of *C. cucurbitarum* were normal. The germ tubes emerged from the central part of the sporangiola and sporangiospore, and the shape of the germ tubes was normal (Figure 7B). Mean germ tube lengths of sporangiola/sporangiospore were shorter than those at the control treatment, which were 5.12/8.45, 3.91/7.37, 9.32/7.54, 7.18/7.23, and 6.37/6.53 μm on PDA containing boscalid, dimethomorph, isopyrazam, propiconazole, and tebuconazole, respectively. On PDA without any fungicide, the germ tube lengths of sporangiola and sporangiospore were 48.29 and 45.65 μm (Figure 7B).

## 4. Discussion

*Choanephora cucurbitarum* was frequently associated with rot on the flower, stem, and leaf of a variety of hosts (Table 1). In the past, Sun et al. also reported that on quinoa stem rot caused by *C. cucurbitarum* in China [14]. The present study showed for the first time that *C. cucurbitarum* could cause the rot of quinoa panicle neck and leaf in China, which could lead to a decrease in yield. Our research indicated that CRC on quinoa first appeared in the panicle neck, and gradually expanded to the stem and leaf. Similarly, findings suggested that *C. cucurbitarum* mostly infected flowers and young fruits [24,30]. Therefore, it is necessary to monitor quinoa panicle neck rot in the field. Quinoa panicle neck rot is the early stage of CRC and also the critical period of disease management. *C. cucurbitarum* mainly infected quinoa panicle neck and stem but could also cause quinoa leaf rot in China. Compared with the leaf rot of other hosts, *C. cucurbitarum* can result in rapid soft rotting of the whole quinoa leaves (Table 1). These results suggest that the infection of quinoa leaves should raise concern and further investigation.

Currently, *C. formosanum* is grown as an ornamental and grain crop and *C. album* is a native weed in China [55]. For the time being, there is no report about the association of *C. cucurbitarum* on the panicle neck and stem of *C. formosanum* and *C. album*. The present study found for the first time that *C. cucurbitarum* is the pathogen causing panicle neck and stem rot of *C. formosanum* and stem rot of *C. album*. It should be noted that CRC symptoms on *C. formosanum* and *C. album* growing adjacent to infected quinoa were observed. We speculate that the host ranges of *C. cucurbitarum* have extended and are likely to continue expanding. Therefore, it is important to reduce further spread of CRC. The proper layout *C. formosanum* and *C. quinoa* and removal *C. album* in the field are essential for control of CRC. Interestingly, CRC is not observed in the panicle necks of *C. album*. It is hypothesized that panicle traits are also probably one of the factors. The lax panicles of *C. album* may keep them in relatively low humidity that is not infected by CRC, especially during periods of rainfall.

Correct diagnosis is a fundamental requirement for effective disease management. As shown in previous studies, the three genes (LSU, ITS, and SSU) for identification of the genus of *Choanephora* have been used for resolution at the species level [27,31,56]. We found that a lot of reference strains were from the CBS culture collection whose SSU were unknown [56]. Because the reference strains had only one or two sequences of the three genes, the phylogenetic tree would be different. Therefore, both ITS and LSU are recommended as the most useful genes for the identification of *Choanephora* species. In future, more phylogenetically informative genes are required to identify the genus of *Choanephora*, especially such as SSU. In this study, the representative isolates (LMJM-2, LMJM-3, LMJM-5, LMJM-7, and LMJM-9) clustered in the same branch as *C. cucurbitarum* based on the analysis of LSU and ITS. The morphological characterization (sporangiola, sporangia, and sporangiospores) of the representative isolates that infected *Chenopodium* plants were generally consistent with the model strain of *C. cucurbitarum*. A set of morphology, molecular characterization, and pathogenicity evaluation identified *C. cucurbitarum* as the pathogen causing CRC. 

The Choanephora diseases frequently occur in tropical and subtropical regions featuring high temperatures and humidity [57]. Our results showed that the developments of CRC were very rapid, with a very short time (1 to 3 days). The germination rates of the sporangiola and sporangiospores of *C. cucurbitarum* were 77.43% and 70.67% by 2 hpi at 30 °C and RH = 75%. Pathogenicity tests showed that *C. cucurbitarum* could infect the panicle necks of *C. quinoa* and *C. formosanum*, and the stems of *C. quinoa*, *C. formosanum*, and *C. album* at 30 °C after 1 dpi. This may also partially explain why CRC outbreaks could appear within a very short time, particularly during high humidity and temperatures. Is has also been reported that the suitable environmental conditions (25 to 30 °C and 70–90% relative humidity) could promote infection of *C. cucurbitarum* [28,31,57,58]. Between July and August in 2022, the weather conditions of *C. formosanum* and *C. quinoa* plantations of Shanxi Province were hot and humid, which were preferred by *C. cucurbitarum* for infections. This also can explain why *Chenopodium* plants are more susceptible to CRC in summer. Noteworthily, there are two kinds of sporangiola and sporangiospores in *C. cucurbitarum*, which indicates the need for targeted control.

Sporangiola and sporangiospores of *C. cucurbitarum* play an important role in early infection, resulting in a rapid spread and devastating loss of CRC. Inhibition germination of sporangiola and sporangiospores is important in the early prevention of CRC. The toxicological effects of the same types of fungicide can differ in the same pathogen [47]. Among the five fungicides in this study, isopyrazam had the strongest inhibitory effects on sporangiola germination. Meanwhile, isopyrazam and tebuconazole had relatively high inhibitory effects against sporangiospore germination. We speculate that isopyrazam and tebuconazole may provide preventive activities to control CRC. Tebuconazole and propiconazole are both triazole fungicides, but the EC_50_s of tebuconazole and propiconazole to *C. cucurbitarum* are different. This may be related to the molecular structure of tebuconazole and propiconazole and may also be related to the targets of tebuconazole and propiconazole in *C. cucurbitarum*. Additionally, boscalid and dimethomorph have moderate inhibitory activities against sporangiospore germination. This demonstrates the potential of boscalid and dimethomorph to combat CRC.

## 5. Conclusions

In conclusion, this firstly reports the occurrence of *C. cucurbitarum* on *Chenopodium* plants based on morphological characteristics, phylogenetic analysis, and pathogenicity analysis in many regions of Shanxi, China. Among the five tested fungicides, isopyrazam showed a higher sensitivity to sporangiola germination of *C. cucurbitarum*. Isopyrazam and tebuconazole strongly inhibited the sporangiospore germination of *C. cucurbitarum*. Therefore, isopyrazam and tebuconazole may provide preventive activities to control CRC. The findings of this study will provide important information on the recognition, diagnosis, and management of these diseases. In the future, surveys of this pathogen are needed to assess its genetic diversity, infection mechanisms, and epidemiology to combat it. Additionally, different strains of *C. cucurbitarum* from different countries and research on the pathogenicity and host range between different strains need the cooperation of researchers.

## Figures and Tables

**Table 1:** The worldwide distribution of the hosts of *Choanephora cucurbitarum*.

Host	Disease	Country	References
*Abelmoschus esculentus*	Blossom blight	Korea	[24]
	Leaf blight	China	[37]
	Pod soft rot	Korea, Bangladesh	[24,38]
	Stem canker	Bangladesh	[38]
*A. manihot*	Blossom blight	Korea	[26]
	Flower wet rot		
*Althaea officinalis*	Flower blight	Korea	[29]
*Brassica chinensis*	Leaf wet rot	Thailand	[39]
*Capsicum annuum*	Blossom blight	United States	[23]
	Fruit soft rot		
	Leaf blight		
	Shoot tip dieback		
*Catharanthus roseus*	Flower blight	United States	[40]
*Carya illinoinensis*	Leaf spot	China	[41]
*Crotalaria spectabilis*	Flower blight	Brazil	[42]
	Stem blight		
*Cucurbita pepo*	Blossom blight	Mexico	[43]
	Fruit soft rot	Slovenia	[44]
*C. moschata*	Blossom blight	Slovenia	[44]
	Fruit soft rot		
*C. quinoa*	Stem rot	China	[14]
*Crotalaria breviflora*	Flower rot	Brazil	[33]
	Leaf wilt		
	Stem necrosis		
*Dahlia pinnata*	Flower blight	Korea	[28]
*Hibiscus syriacus*	Flower rot	Korea	[27]
*Hosta plantaginea*	Flower wet rot	Korea	[25]
*Hyoscyamus muticus*	Floral tops rot	Japan	[31]
*Lactuca sativa*	Leaf rot	Korea	[34]
*Mesembryanthemum crystallinum*	Leaf rot	Japan	[32]
	Stem rot		
*Moringa oleifera*	Seed pod rot	China	[36]
*Petunia hybrida*	Flower blight	United States	[45]
	Flower wet rot		
*Phaseolus vulgaris*	Blossom blight	United States	[23]
	Fruit soft rot		
	Leaf blight		
	Shoot tip dieback		
*Pinellia ternata*	Flower blight	China	[30]
	Leaf rot		
	Stem rot		
*Ricinus communis*	Seedling rot	China	[22]
*Solanum melongena*	Soft rot	Korea	[35]
*Withania somnifera*	Leaf wet rot	India	[46]
	Stem wet rot		

**Table 2:** Names, strain numbers and corresponding GenBank accession numbers of the taxa used for phylogenetic analyses. ^T^—Ex-type strains.

Species	Strain Number	GenBank Accession Number	
		LSU	ITS
*C. cucurbitarum*	LMJM-2	OR002181	OR002157
	LMJM-3	OR002182	OR002158
	LMJM-5	OR002183	OR002159
	LMJM-7	OR002184	OR002160
	LMJM-9	OR002185	OR002161
	CBS 178.76^T^	MT523842	JN206235
	CBS 674.93	JN939195	JN206233
	JPC1	MH041504	MH041502
	JSAFC2346	OP315251	OP315248
	JSAFC2347	OP315252	OP315249
	JSAFC2348	OP315253	OP315250
	KA47637	KJ461160	KJ461159
	KA47639	KJ461162	KJ461161
	KUS-F27485	KR867729	KR867728
	KUS-F27538	KP726892	KP726891
	KUS-F27540	KM200035	KM200034
	KUS-F27657	KR867731	KR867730
	KUS-F28029	KT581013	KT581012
	KUS-F28066	KP406600	KP406599
	KUS-F29113	KU316935	KU316934
	QJFY1	MW341527	MW295532
*C. infundibulifera*	CBS 153.51	JN939193	JN206236
	KUS-F27535	KJ486538	KJ486539
	KUS-F28085	KR867733	KR867732
	KUS-F28106	KR867735	KR867734
*Blakeslea trispora*	CBS 564.91^T^	JN206515	JN206230

**Table 3:** Concentration of the five fungicides used in this study.

Fungicide	Concentration (μg/mL)	
	Sporangiola Germination	Sporangiospore Germination
Tebuconazole	0.5, 1, 5, 10, 20	0.1, 0.15, 0.2, 0.5, 1
Propiconazole	1, 25, 50, 75, 100	5, 7.5, 10, 15, 20
Boscalid	10, 15, 20, 40, 60	0.5, 0.75, 1, 1.5, 2
Isopyrazam	0.1, 0.5, 1, 1.5, 2	0.1, 0.25, 0.35, 0.5, 1
Dimethomorph	10, 15, 20, 25, 30	0.5, 0.75, 1, 2, 2.5

## Data Availability

All data generated or analyzed in the study are available from the corresponding author upon reasonable request.
